# Synergistic Optimisation of Surface Properties and Surface Quality of FDM ABS Specimens Based on RSM and CWOA

**DOI:** 10.3390/polym18151924

**Published:** 2026-08-05

**Authors:** Jing Zhao, Rui Zhu, Hairui Ma, Xinyan Li, Li Yang, Pei Li, Shuangjun Wang, Tianlu Wei

**Affiliations:** 1 School of Mechanical and Vehicle Engineering, Bengbu University, Bengbu 233030, China; zhurui@bbc.edu.cn (R.Z.); 18551782760@163.com (H.M.); 15755635906@163.com (X.L.); yang1155@bbc.edu.cn (L.Y.); 13305522301@163.com (S.W.); wtltong@163.com (T.W.); 2 Anhui Province Additive Manufacturing Engineering Research Center, Bengbu 233030, China

**Keywords:** FDM, ABS, chaotic whale optimisation algorithm, Ra, COF, synergistic optimization

## Abstract

The surface roughness (Ra) and coefficient of friction (COF) of acrylonitrile butadiene styrene (ABS) parts fabricated through fused deposition modelling (FDM) are key determinants of their functional service performance. However, these two objectives often present a trade-off relationship in single-objective optimisation. To achieve synergistic optimisation of surface quality and surface performance of FDM ABS parts, this paper proposes a multi-objective optimisation framework integrating response surface methodology (RSM) with a chaotic whale optimisation algorithm (CWOA). A Box–Behnken design (BBD) was employed to establish quadratic regression models for Ra and COF as functions of layer thickness (0.16–0.24 mm), extrusion ratio (0.9–1.0), infill density (20–100%) and extrusion temperature (240–270 °C). Based on analysis of variance (ANOVA) and response surface analysis, a non-linear mathematical model with dual responses was constructed. Pareto-dominated CWOA was introduced for multi-objective optimisation, and the optimal process parameter combination was selected using the TOPSIS method. The results showed that the optimal parameters were: layer thickness 0.16 mm, extrusion ratio 0.9, infill density 66%, and extrusion temperature 270 °C. Under these conditions, the experimentally measured Ra was 7.7921 μm (2.04% error from the predicted value) and COF was 0.1504 (2.80% error from the predicted value). Compared with the benchmark reference (the average value of the BBD central experimental runs), a synchronous decrease in Ra and COF was achieved (Ra reduced by 27.4% and COF reduced by 14.9%). Metallographic morphology analysis revealed that the optimal specimen surface exhibited wide filament ridges, narrow and well-defined inter-filament valleys, and no obvious forming defects, achieving synergistic low roughness and low friction. The proposed RSM-CWOA framework provides an effective method for multi-objective optimisation of FDM processes and can be extended to other polymer additive manufacturing systems.

## 1. Introduction

Among the various currently available additive manufacturing (AM) methods, fused deposition modelling (FDM) dominates both desktop and industrial-scale polymer printing, primarily due to its low capital cost, high material utilisation, and direct conversion of digital models into physical objects [1,2,3]. Originally developed as a rapid prototyping method, FDM has evolved into a production-grade technology capable of manufacturing functional end-use parts—including jigs, fixtures and structural components—for the aerospace, automotive, biomedical and consumer electronics sectors [4,5]. This process deposits thermoplastic filament in a layer-by-layer manner through a heated nozzle. Compared with subtractive or other shaping methods, this additive mechanism offers two inherent manufacturing advantages: the ability to achieve virtually unlimited geometric complexity without dedicated tooling and a substantially reduced time to market. However, this layerwise construction inevitably introduces characteristic defects such as the “staircase effect” [6], interlayer voids and porosity [7], all of which affect surface integrity, dimensional accuracy and mechanical properties of printed parts. Consequently, process parameter optimisation has become a central theme in FDM research.

Among the various thermoplastics suitable for FDM, acrylonitrile butadiene styrene (ABS) is one of the most widely used engineering materials due to its excellent mechanical properties, and it is primarily employed in the manufacture of structural parts, housing, automotive interior components, consumer electronic casings and functional elements [8]. However, the thermal and rheological characteristics of ABS also render it highly sensitive to operating conditions; inappropriate parameter selection can lead to poor interlayer bonding, excessive warpage and pronounced surface defects, thereby affecting the reliability of ABS parts and composite ABS filament parts in demanding applications [9,10].

For ABS parts intended for functional applications, two surface-related performance indicators are critical: surface roughness (Ra) and coefficient of friction (COF). Ra, typically quantified as the arithmetic mean deviation of the surface profile, directly affects the appearance, fatigue resistance, wettability, coating adhesion and local stress of printed parts. In assemblies where parts must fit precisely or withstand cyclic loading, even moderate changes in roughness can induce premature failure [11]. Therefore, Ra in FDM ABS is not merely an aesthetic attribute but a key determinant of mechanical reliability. COF is a physical quantity describing the frictional resistance between objects, and it determines frictional performance, energy dissipation, noise generation and overall service life, particularly in sliding or contacting applications such as gears, bearings, snap-fit assemblies and sliding guides [12,13]. The frictional performance of additively manufactured polymers has been identified as one of the greatest challenges for functional deployment, especially in load-bearing applications where frictional performance is a critical concern [14]. Studies have begun to explore the complex relationship between surface topography and frictional performance. Recent work by Mahmood et al. [15] conducted experiments on FDM-printed polylactic acid (PLA) and found that skewness (Ssk) and maximum valley depth (Sv) exhibited stronger positive correlations with COF than average surface roughness (Ra), indicating that extreme topographical features, rather than average roughness, dominated the frictional response. Portoacă et al. [16] analysed the effects of process parameters on the coefficient of friction, surface roughness parameters and cumulative linear wear of 3D-printed PLA and ABS parts. Their results showed that, under the same layer thickness and infill density, the surface roughness of ABS parts was consistently higher than that of PLA parts; however, the comparison of COF and cumulative linear wear between the two materials depended on the specific process parameters: under some parameter sets ABS parts exhibited lower values, while under others PLA parts were lower. Taken together, these findings indicate that the unique layerwise structure of FDM produces a complex, anisotropic surface texture, resulting in a relationship between Ra and COF that is neither monotonic nor independent, and there is no simple one-to-one correspondence between the two. However, the simultaneous collaborative optimisation of Ra and COF within a unified modelling and optimisation framework remains insufficiently explored in existing research.

Considerable research efforts have been dedicated to understanding and optimising the effects of FDM process parameters on surface quality and mechanical properties. The influence of key deposition variables such as layer thickness (LT), extrusion temperature (ET), scan path, infill density (ID) and printing speed on dimensional accuracy [17,18,19,20,21] and mechanical properties [22,23,24,25,26] has been systematically investigated. Mushtaq et al. [27] adopted central composite design to explore the single-objective responses of ABS parts, and reported that increased layer thickness significantly elevates surface roughness Ra, while moderate infill density balances flexural strength and surface smoothness; however, the work ignored the variation trend of friction coefficient (COF) under identical parameter combinations. Ouazzani et al. [28] focused only on Ra of ABS specimens and confirmed that minimum LT (0.1 mm) and maximum ET (260 °C) produce the lowest Ra, yet the corresponding tribological performance was not characterised. Mohan et al. [29] utilised Box–Behnken design to reveal that Ra rises with increasing infill density, and the interaction between bed temperature and layer thickness dominates surface roughness fluctuation, but the friction behaviour of printed surfaces was not analysed. Sagar et al. [30] comparatively investigated PLA and ABS across full infill density gradients (0–100%) and found that rising infill density increases microhardness of ABS, while Ra gradually declines with denser internal filling; nevertheless, the coupled evolution rule of COF and Ra under coordinated changes in LT, ET and ER was not systematically quantified. Though these studies clarify the individual influence of single parameters on surface quality or mechanical performance, few reports simultaneously track the coupled variation in Ra and COF under the joint action of LT, ER, ID and ET, and the competitive relationship between low roughness and low friction has not been revealed.

In many real-world application scenarios—such as sliding guides, snap-fit assemblies and automotive interior parts—a component must simultaneously satisfy requirements for low friction (to reduce wear and energy loss) and high surface quality (to ensure proper fit, aesthetics and fatigue resistance). However, achieving these two objectives simultaneously is challenging, because process parameters that favourably reduce surface roughness may be detrimental to friction reduction, and vice versa. To address this challenge, metaheuristic optimisation algorithms offer a promising route by enabling exploration of large, non-linear, multimodal search spaces without requiring gradient information or subjective weighting of responses. The whale optimisation algorithm (WOA), originally proposed by Mirjalili et al. [31], is a bio-inspired metaheuristic based on the hunting behaviour of humpback whales, and it is valued for its simplicity and effective exploration–exploitation balance. However, standard WOA suffers from slow convergence and a tendency to become trapped in local optima when applied to complex, high-dimensional problems. To overcome these limitations, Kaur and Arora introduced chaos theory into WOA, proposing the chaotic whale optimisation algorithm (CWOA), which uses chaotic maps to replace key parameters controlling local and global search, significantly improving convergence speed and global optimum reliability [32]. The strategy of enhancing metaheuristic algorithms with chaos has been validated in various engineering design optimisation problems [33]. Regarding multi-objective optimisation, response surface methodology (RSM) has been widely adopted as an efficient statistical modelling and optimisation technique [34,35]. RSM has been used for optimisation in various contexts [36]. RSM may be more suitable for optimising multi-response 3D printing because it can model with higher fidelity and multi-objective capability [37].

In response to the research gap on the coupled evolutionary relationship and collaborative optimisation of surface roughness (Ra) and coefficient of friction (COF) in fused deposition modelling (FDM)-fabricated acrylonitrile butadiene styrene (ABS), this study constructs a multi-objective collaborative optimisation framework combining response surface methodology (RSM) and chaotic whale optimisation algorithm (CWOA). We systematically investigate the independent and interactive effects of four key process parameters—layer thickness (LT), extrusion ratio (ER), infill density (ID), and extrusion temperature (ET)—on the surface roughness and friction coefficient of FDM-printed ABS specimens. The inherent coupling mechanism linking surface morphology and friction performance is revealed, and a trade-off optimal parameter combination is finally derived to achieve synchronous reductions in Ra and COF. The modelling, optimisation and characterisation approaches proposed in this work can be further extended to multi-objective parameter optimisation for other polymer additive manufacturing systems.

## 2. Materials and Methods

### 2.1. Printing Equipment and Printing Materials

An FDM desktop 3D printer (Model P2S, Bambu Lab Technology Co., Ltd., Shenzhen, China) was adopted for specimen fabrication. ABS filament supplied by Tuozhu Technology Co., Ltd., Shenzhen, China. was utilised, with a density of 1.05 g/cm^3^ and a diameter of 1.75 mm. Its key thermal properties included a Vicat softening temperature of 94 °C, a heat deflection temperature of 87 °C, and a melting temperature of 200 °C. Featuring high toughness, good mechanical durability and favourable thermal stability, the filament was vacuum-dried before printing to remove internal moisture. Two types of specimens were fabricated separately for friction wear tests and surface roughness measurements. The friction and wear specimens were cylindrical with dimensions of Φ25 × 10 mm^3^. The specimens for roughness testing measured 50 × 15 × 10 mm^3^; their 50 mm length was aligned with the *X*-axis of the printing platform, and the 15 mm width was parallel to the *Y*-axis.

### 2.2. Characterisation Methods

**Figure 1:**
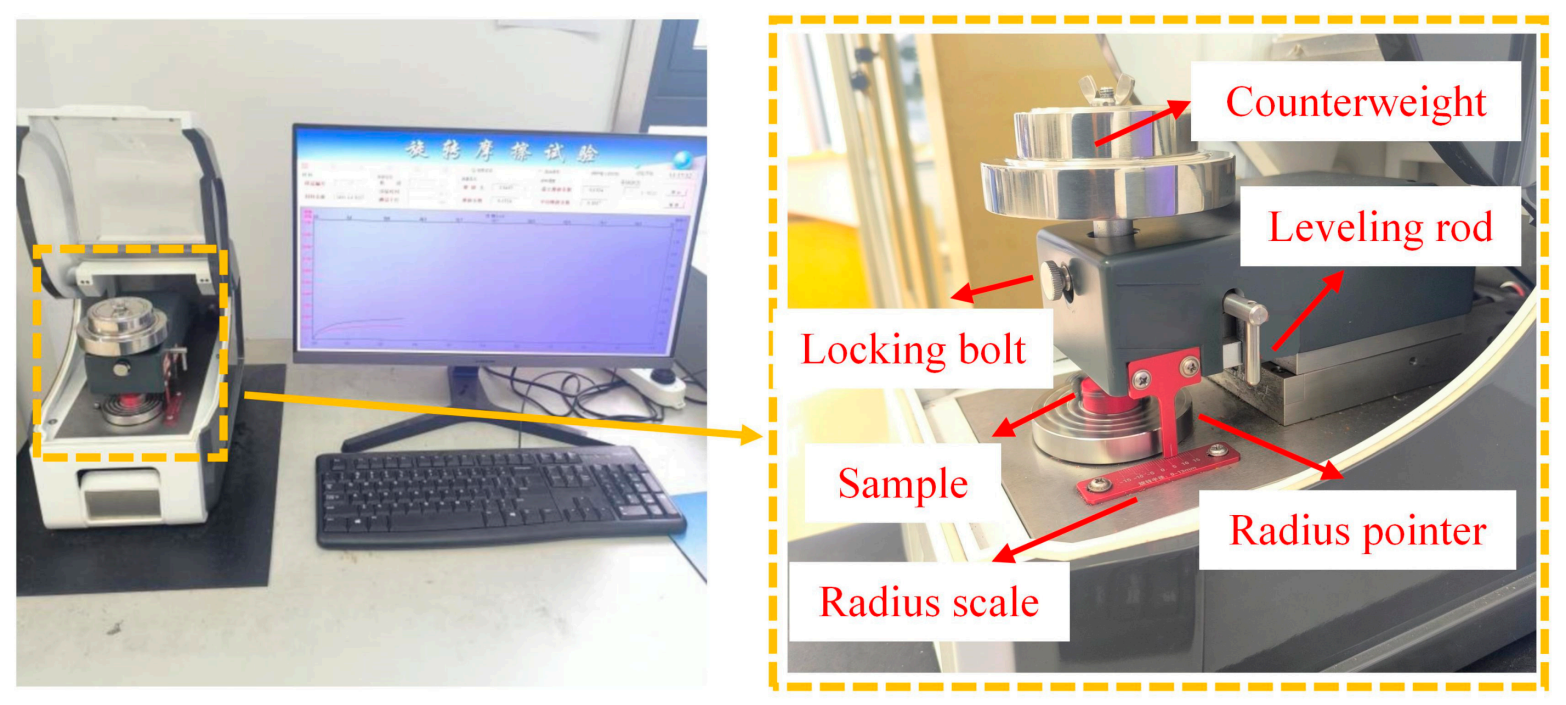
Friction and wear tester.

Friction and wear performance was characterised via an MS-T3100 tribometer (Lanzhou Huahui Instrument Technology Co., Ltd., Lanzhou, China), as illustrated in Figure 1. All tribological tests were conducted under ambient conditions of 23 ± 2 °C and 50 ± 10% relative humidity. GCr15 bearing steel balls (Jiada Steel Ball, Suzhou, China)were used as the friction balls, with a diameter of 5 mm and a hardness of 60–63 HRC. The initial surface roughness Ra was approximately 0.02 μm. The steel ball rotated against the top surface of each printed specimen, with a wear track radius of 4 mm, rotational speed of 300 RPM, test duration of 900 s, and applied normal load of 15 N. For each specimen, the average value of instantaneous friction coefficients recorded over the full test cycle was adopted as the final COF index. This processing method avoids systematic errors caused by artificial interception of test data. COF was calculated according to Equation (1):

(1) COF=F/N

where COF is the coefficient of friction, *F* is the friction force (N), and *N* is the normal load (N).

**Figure 2:**
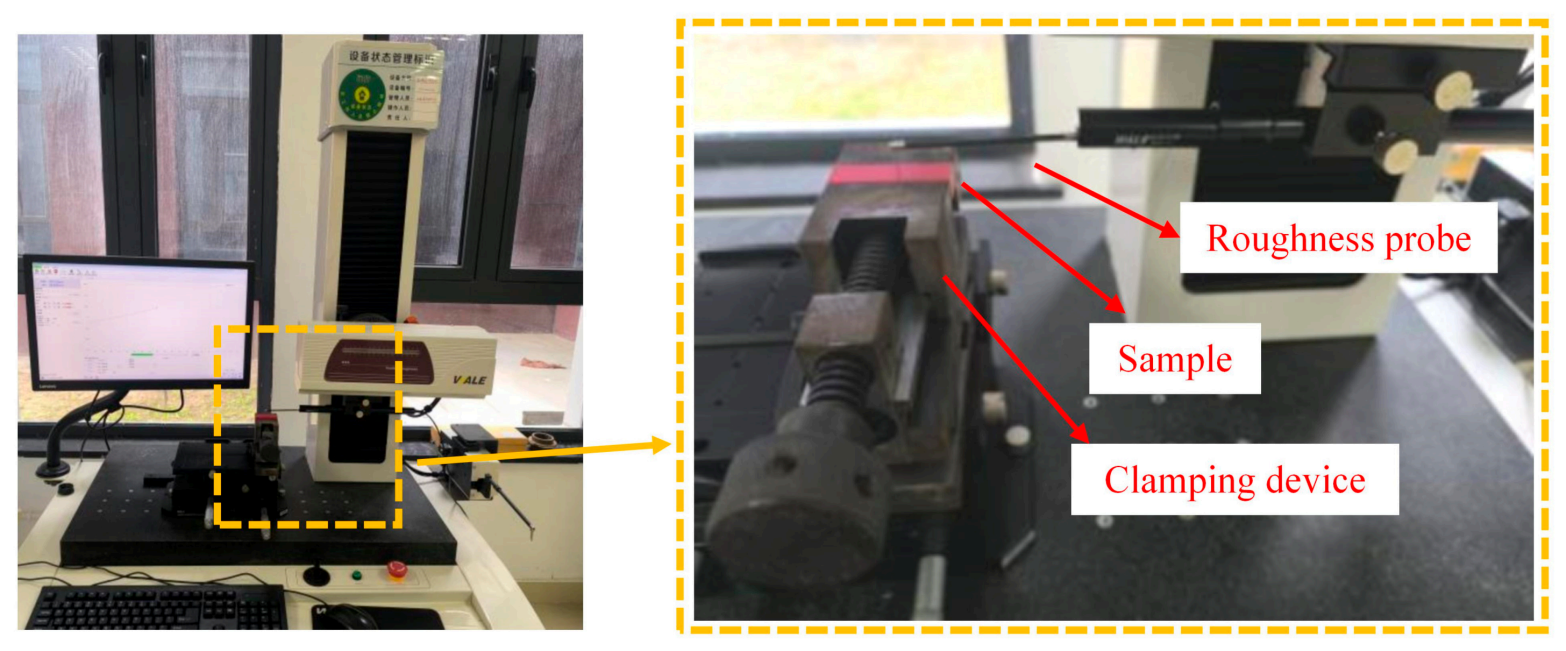
Roughness and contour integrated instrument.

Roughness was characterised using an SP2012W-tco roughness and contour integrated machine (Wale Electromechanical Technology Co., Ltd., Xi’an, China) as shown in Figure 2. Contact contouring was used to characterise the arithmetic mean roughness Ra. The roughness measurement needle has a tip radius of 25 μm, a stylus force of 75 μN, a measurement speed of 0.2 mm/s, a sampling interval of 0.1 μm, a cutoff wavelength filtering of 8 mm, and a sampling length of 32 mm. The stylus travelled along the longitudinal X-direction of the specimen surface at a 45° angle to the printed filament tracks. Five parallel measuring lines were evenly arranged along the Y-direction on the top surface of each specimen. After eliminating the maximum and minimum Ra values, the average of the remaining three measurements was taken as the final roughness value for each sample. Ra was calculated using Equation (2):

(2) $$\mathrm{Ra}=\frac{1}{L}\int \begin{array}{c} L\\ 0 \end{array}\left|y(x)\right|dx$$

where Ra is the arithmetic mean deviation of the profile (µm), *L* is the evaluation length (mm), and *y*(*x*) is the vertical deviation of a point on the profile curve from the mean line (µm).

### 2.3. Experimental Design

**Figure 3:**
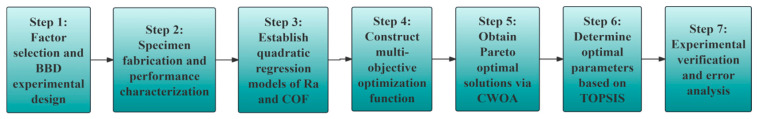
Multi-objective optimisation experimental flowchart based on BBD-CWOA-TOPSIS.

The multi-objective optimisation experimental process based on BBD-CWOA-TOPSIS was shown in Figure 3. The experiment was based on the BBD experimental design method in response surface methodology, exploring the effects of four main process parameters LT, ER, ID, and ET on the Ra and COF of the samples at three levels, with a total of 29 experiments. The regression equations for Ra and COF were then combined to construct a multi-objective function. Subsequently, the chaotic whale optimisation algorithm (CWOA) was used to optimise the multi-objective function to obtain optimal process parameters and predicted response values. The TOPSIS method was employed to comprehensively evaluate the multi-objective optimisation results. Finally, specimens were printed using the optimal process parameters to validate the error between predicted and experimentally measured response values.

## 3. Results and Analysis

### 3.1. RSM-BBD Experimental Design and Factor Level Determination

Box–Behnken design (BBD) is an efficient response surface methodology with fewer experimental runs, uniform factor distribution and built-in central points, suitable for fitting quadratic regression models and multi-factor exploratory optimisation, which has been widely adopted in manufacturing experimental research.

Four core FDM parameters (LT, ER, ID, ET) were selected. The definitions of each parameter are given as follows: LT refers to the height of each layer in the printed model; ER is defined as the ratio of the actual molten material volume extruded from the nozzle along a given printing path to the theoretical target extrusion volume calculated by slicing software; ID denotes the internal infill density; ET represents the nozzle extrusion temperature. All other slicing and printing settings were kept constant: a 0.4 mm nozzle, 0.42 mm line width, printing speed of 200 mm/s, four top/bottom solid layers and four outer perimeters, linear infill pattern, ±45° alternating outer raster angle, 100 °C build plate temperature, and 0% cooling fan speed.

Based on the above fixed printing device and the ABS filament used, multiple extreme combinations of LT, ER and ID were tested to narrow down valid parameter ranges capable of fabricating dimensionally stable specimens without warpage, delamination or nozzle clogging. Parameter combinations outside these ranges either interrupted printing halfway or induced severe manufacturing defects. The extrusion temperature range of 240–270 °C was selected based on the processing window recommended by the filament supplier. Temperatures below 240 °C yielded only partial melting and failed to generate a continuous molten stream suitable for printing, whereas temperatures above 270 °C initiated thermal degradation of ABS. Excessively high LT, ER or ID values caused melt overflow and irregular top surfaces; conversely, overly low values led to insufficient melt feeding and poor internal structural support. The selected range (LT 0.16–0.24 mm, ET 240–270 °C, ID 20–100%, ER 0.9–1.0) represents the fully printable interval without fatal printing failure. However, local micro-morphology imperfections (local incomplete melting micro-pits, disordered inter-filament valleys) may still exist within this stable window, which dominate the variation in Ra and COF, rather than causing print abortion. The experimental parameters and their corresponding coded levels are listed in Table 1. In the BBD, the minimum, intermediate, and maximum values of each factor were designated as −1, 0, and +1, respectively.

### 3.2. BBD Experimental Results

Based on the BBD criteria, 29 experimental runs were conducted with four factors and three levels. The specific experimental design parameters and results are presented in Table 2. COF reflects the frictional performance of the specimen; a lower COF indicates better surface frictional performance [38]. Ra reflects the surface roughness; a lower Ra indicates better surface smoothness [39].

#### 3.2.1. Analysis of Variance (ANOVA)

The ANOVA results for COF are shown in Table 3. The regression model exhibited a highly significant predictive effect for COF (F = 18.03, *p* < 0.0001), with a coefficient of determination R^2^ of 0.9474 and an adjusted R^2^ of 0.8949, indicating that the model explained approximately 94.74% of the variation in COF. Single-factor effects analysis showed that factor A had a highly significant impact on COF (F = 17.79, *p* = 0.0009), and factor B also had a highly significant impact (F = 23.63, *p* = 0.0003), which is consistent with the results reported in Refs. [16,40]. In terms of interaction effects, the AC (LT-ID) interaction term was highly significant (F = 58.66, *p* < 0.01), the AD (LT-ET) interaction term was highly significant (F = 11.69, *p* < 0.01), the BC (ER-ID) interaction term was highly significant (F = 47.49, *p* < 0.01), and the BD (ER-ET) interaction term was highly significant (F = 80.66, *p* < 0.01), while the CD (ID-ET) interaction term was highly significant (F = 9.35, *p* < 0.01). The lack-of-fit test for the model was not significant (F = 1.28, *p* = 0.4374), confirming that the model did not exhibit significant lack of fit and that the statistical inferences were reliable.

The ANOVA results for Ra are also presented in Table 3. The model exhibited a high explanatory power for Ra, with a highly significant regression model (F = 16.28, *p* < 0.01), an R^2^ of 0.9421, and an adjusted R^2^ of 0.8843, indicating good model fit and predictive performance. For single-factor effects, factor B (ER) had a highly significant effect on Ra (F = 120.08, *p* < 0.01), and factor C (ID) had a highly significant effect (F = 12.49, *p* = 0.0033), both of which are consistent with the results reported in Refs. [16,41]. Among interaction effects, AB (LT-ER) and BC (ER-ID) were significant (F = 8.11 and 4.98, both *p* < 0.05). The model lack-of-fit test for the model was not significant (F = 0.9493, *p* = 0.5721), indicating good agreement between the model and the measured data and confirming that the model could effectively characterise the influence of the process factors on Ra.

**Figure 4:**
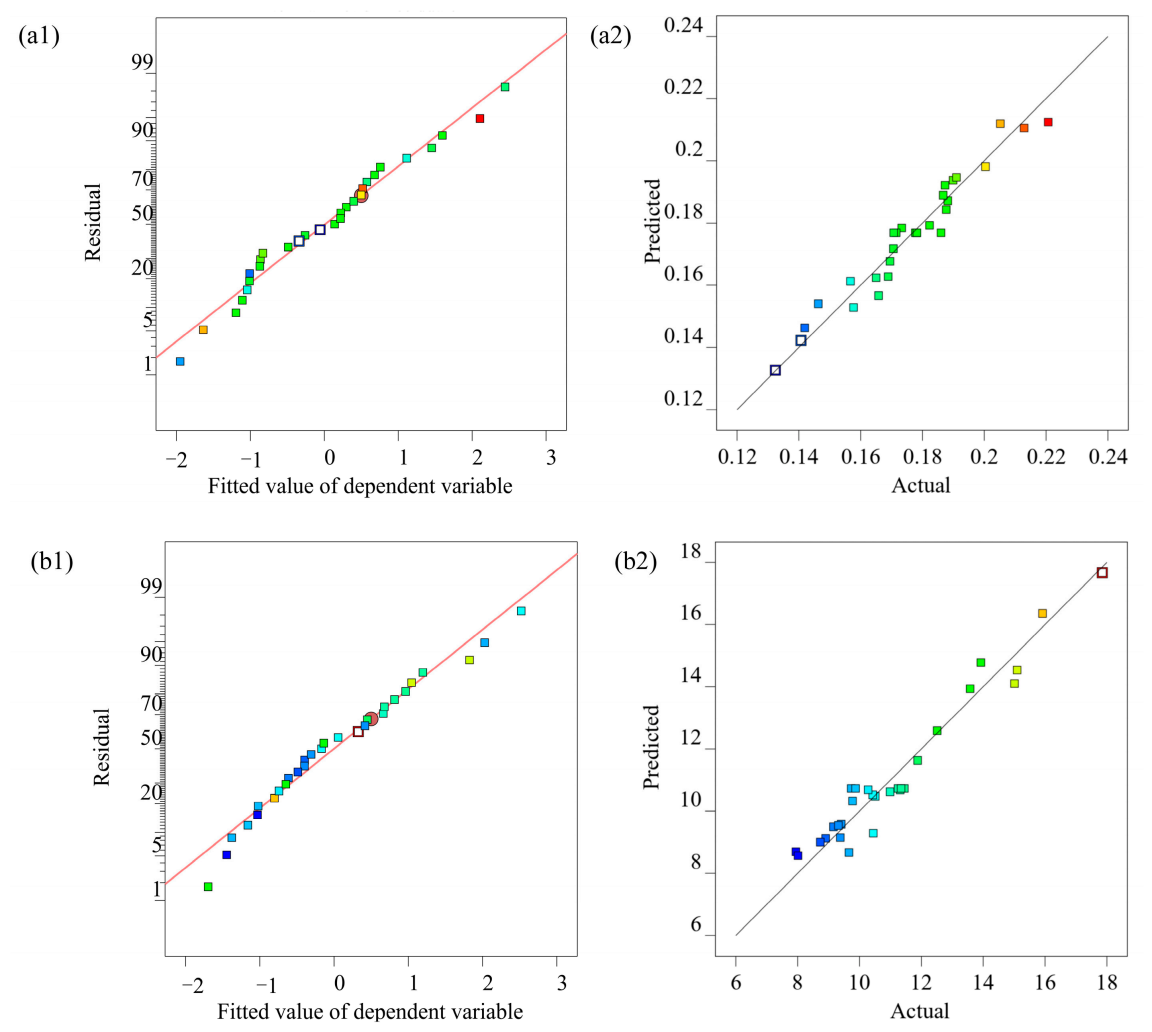
Numerical model analysis. (**a**) Numerical model analysis for COF: (**a1**) residual plot, (**a2**) regression plot. (**b**) Numerical model analysis for Ra: (**b1**) residual plot, (**b2**) regression plot.

Numerical model analyses are shown in Figure 4. Figure 4a presents the numerical analysis for COF and Figure 4b for Ra. The residuals of the experimental results were distributed approximately along a straight line, indicating that the standardised residuals followed a normal distribution. In the plots of predicted versus actual response values, the points were distributed near the straight line, further demonstrating the validity and accuracy of the regression equations. The regression equations in terms of actual factors are given in Equations (3) and (4). Equation (3) is the regression equation for COF, where Z_COF_ represents the COF response; Equation (4) is the regression equation for Ra, where Z_Ra_ represents the Ra response.

Z_COF_ = 9.16431 − 5.31885*A* − 8.89515*B* + 0.012193*C* − 0.035232*D* − 0.912500*AB* + 0.016203*AC* + 0.019292*AD* − 0.011662*BC* + 0.040533*BD* − 1.725 × 10^−5^*CD* + 0.220313*A*^2^ − 0.394000*B*^2^ + 7.90625 × 10^−7^*C*^2^ − 1.187778 × 10^−5^*D*^2^ (3)

Z_Ra_ = 328.95533 + 702.15948*A* − 1607.68922*B* + 0.442667*C* + 2.62962*D* − 594.75000*AB* − 0.420109*AC* − 0.223625*AD* − 0.465887*BC* + 0.331567*BD* + 0.000173*CD* − 119.84323*A*^2^ + 906.78033*B*^2^ + 0.000154*C*^2^ − 0.005683*D*^2^ (4)

#### 3.2.2. Response Surface Analysis for COF

Response surface methodology, by constructing a quadratic regression model between process parameters and responses, uses three-dimensional surfaces and two-dimensional contour plots to visually present the influence of interactions between parameters on COF and Ra of FDM-printed ABS. Based on the ANOVA results above, response surface plots are presented for the five highly significant interaction terms (*p* < 0.01) for COF and the two significant interaction terms (*p* < 0.05) for Ra to analyse the trends and underlying process mechanisms of response variation under parameter interactions. For all response surface plots, non-analysed parameters are fixed at their centre levels (level 0 in Table 1) to ensure uniqueness and accuracy of the analysis.

**Figure 5:**
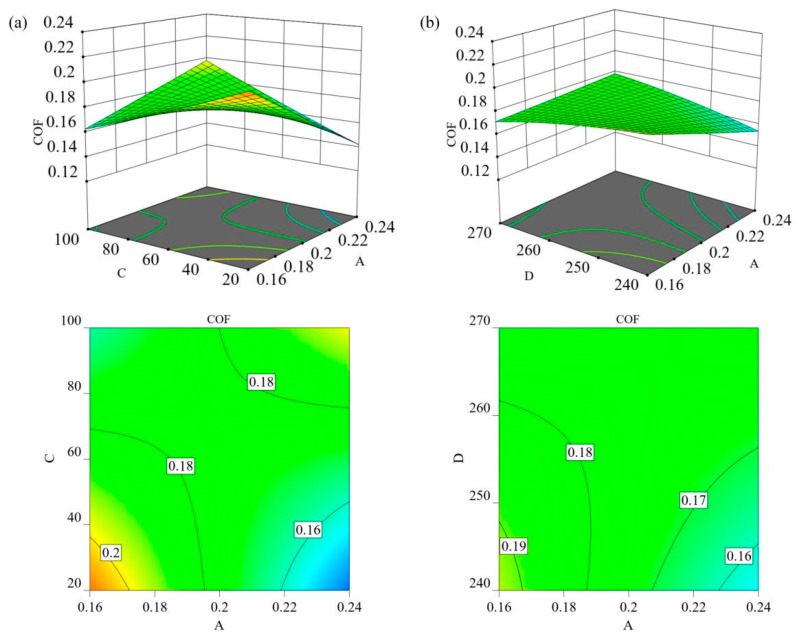

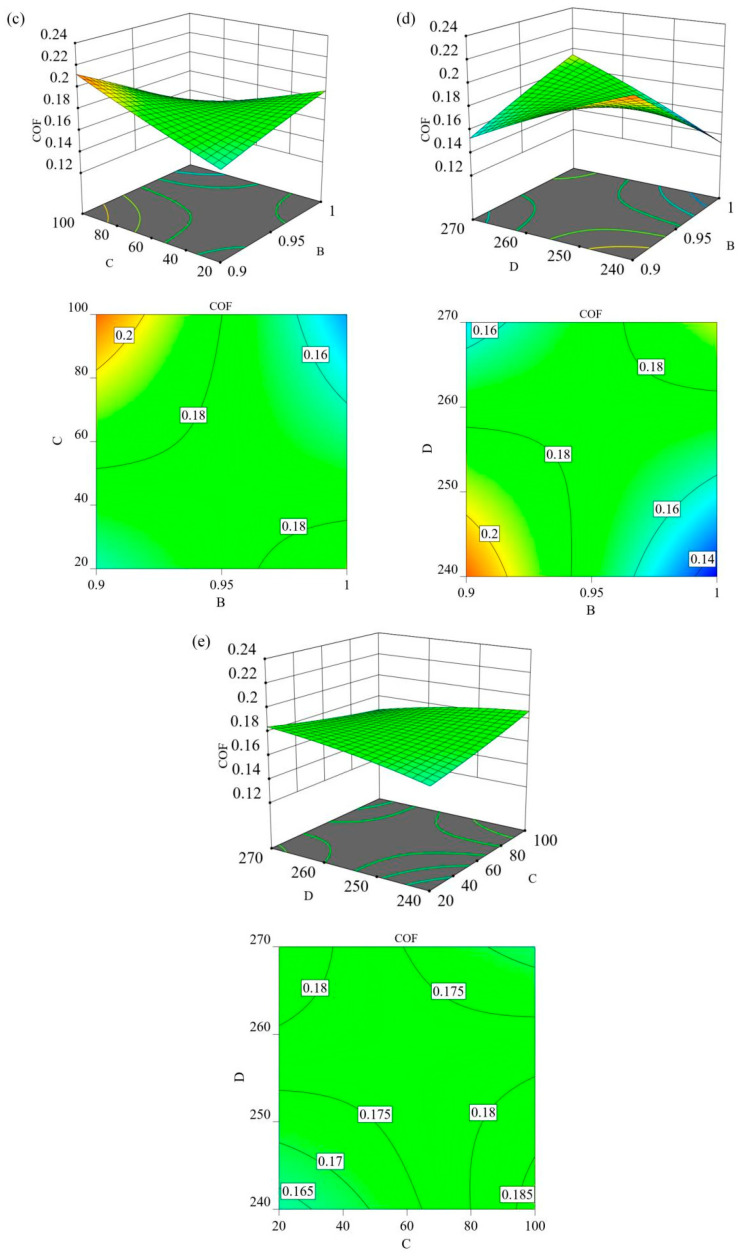
Response surface plots of significant interaction factors affecting COF: (**a**) AC (LT–ID); (**b**) AD (LT–ET); (**c**) BC (ER–ID); (**d**) BD (ER–ET); (**e**) CD (ID–ET).

Figure 5a shows that the AC (LT-ID) interaction has a highly significant effect on COF (*p* < 0.0001), which is similar to the conclusion in Ref. [42]. At low ID (20%), the COF decreased significantly as LT increased from 0.16 mm to 0.24 mm; at high ID (100%), COF continuously increased with increasing LT. This is because under low ID, the internal support strength of the sample is weak, and increasing LT can enhance the surface step effect. This is because the formed inter-filament valleys (groove structures formed between adjacent areas on the surface of the workpiece due to the characteristic of layer-by-layer stacking) can effectively accommodate abrasive particles, which may suppress three body wear [43]. Meanwhile, the gentle contour weakens the hard filament ridges (due to the characteristic of layer-by-layer stacking, the strip-shaped protrusions or textures formed on the surface of the workpiece along the printing path), which may reduce the ploughing effect and lower the COF. At high ID, the interior of the matrix has ample support, the wear-reducing effect of the staircase effect is diminished, which is consistent with the conclusion in Ref. [44], and increased LT tends to cause uneven interlayer cooling, generating disordered valleys, micro-pit defects, and an increase in COF.

Figure 5b shows that the AD (LT-ET) interaction is highly significant (*p* = 0.0041), with a relatively gentle response surface without sharp extrema, which is similar to the conclusion in Ref. [45]. At low ET (240 °C), COF slightly decreased with increasing LT; at high ET (270 °C), COF slightly increased with increasing LT. This is attributed to the relatively insufficient melting degree of the material under low ET compared to high ET conditions within the experimental range; a thin LT tends to produce interlayer bonding defects and micro-delamination, which may be accompanied by significant ploughing. Increasing LT improves interlayer contact, reducing COF. At high ET, the material has good fluidity, interlayer bonding is adequate, and the defect influence of thin LT is eliminated; however, increasing LT introduces internal stresses due to uneven cooling and contraction, making the surface prone to micro-cutting, and COF increases slightly.

In Figure 5c, the BC (ER-ID) interaction is highly significant (*p* < 0.0001), with a saddle-shaped contour distribution. At low ER (0.9), COF significantly decreased as ID decreased; at high ER (1.0), COF significantly decreased as ID increased, which is similar to the results reported in Ref. [46]. This is attributed to the relatively insufficient material supply of the material under low ER conditions compared to high ER conditions within the experimental scope, and high ID exacerbates internal overlap defects, form uneven surface ridges, intensify interface adhesion, and result in high COF. Under high ER, the material is abundant, but a low ID substantially reduces the stiffness of the underlying support structure. During cooling, the excess melt deposited onto this compliant substrate is prone to non-uniform shrinkage and localised micro-settling, generating surface ripples and irregular ridges. More importantly, because the top solid skin layers are thin (six layers) and the infill beneath is porous and flexible, the entire substrate may undergo differential shrinkage and micro-deformation, resulting in macroscopic bending deformation of the upper solid surface. These surface undulations and form deviations may increase the real contact area and ploughing resistance under the spherical probe, thereby elevating the friction force. Appropriately increasing the infill density provides a more uniform and rigid substrate support, which regularises the filament ridge arrangement, stabilises the surface contact conditions, and consequently reduces the COF.

In Figure 5d, the BD (ER-ET) interaction is highly significant (*p* < 0.0001). At low ET (240 °C), as ER increased from 0.9 to 1.0, COF decreases from approximately 0.20 to 0.14; at high ET (270 °C), the modulating effect of ER on COF was significantly weakened, and COF gradually increased with increasing ER, with a relatively gentle response surface. Due to the poor melting of materials at low ET compared to high ET, low ER can lead to insufficient material filling, weak interlayer bonding, and possible exposed pores on the surface, resulting in high COF. Increasing ER improves interlayer bond strength and reduces surface defects, significantly decreasing COF. At high ET, the material has good fluidity and adequate interlayer bonding; the influence of ER on interface strength is diminished, so COF changes relatively gently. However, an excessively high ER may cause material overflow, surface stringing and local material accumulation, thereby increasing the COF, which is consistent with the findings in Ref. [47].

In Figure 5e, the CD (ID-ET) interaction is highly significant (*p* = 0.0085), with a generally gentle response surface and nearly parallel contours. This observation is similar to the content recorded in Ref. [47]. COF fluctuated slightly within the range of 0.165–0.185, without pronounced extrema. ID primarily controls the matrix support within the strands, while ET affects the melt compaction density and defect extent. Their interaction can slightly alter the surface contact state: under high ID and high ET, matrix density increases, the surface morphology becomes uniform and stable, and COF is minimised; under low ID and low ET, profile undulations slightly increase, so COF changes only slightly. Compared with the AC, BC and BD interactions, the CD interaction has a smaller modulating effect on the friction contact mechanism, but still exerts a significant and regular influence on COF.

#### 3.2.3. Response Surface Analysis for Ra

**Figure 6:**
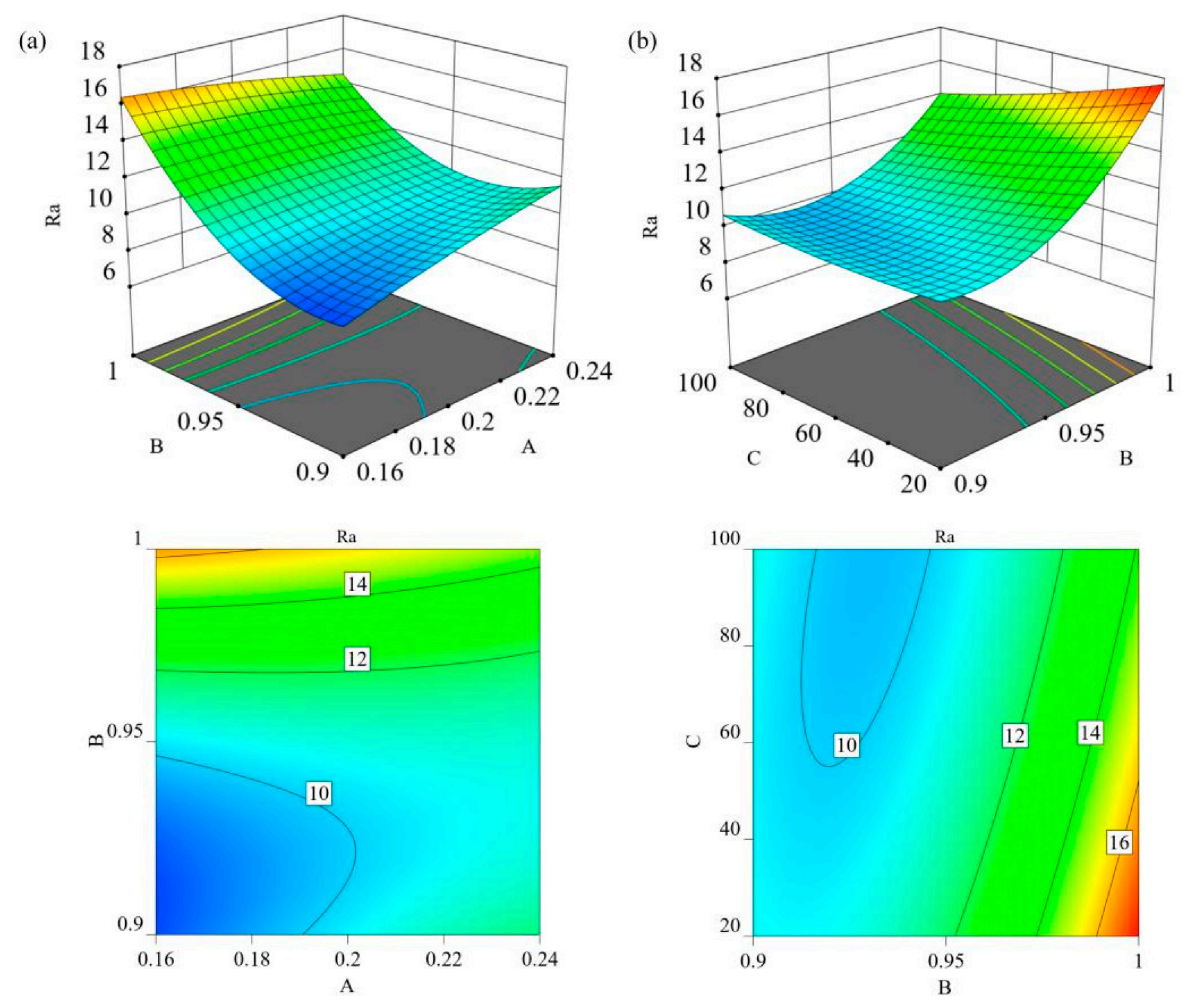
Response surface plots of significant interaction factors affecting Ra: (**a**) AB (LT–ER); (**b**) BC (ER–ID).

In Figure 6a, the AB (LT-ER) interaction has a significant effect on Ra (*p* = 0.0129). At low ER (0.9), Ra significantly decreased with decreasing LT, reaching the lowest value in the LT range of 0.16–0.18 mm; at high ER (1.0), Ra slowly increased with decreasing LT. This observation is similar to the results recorded in Ref. [48]. This phenomenon arises because, at low ER, the supply of molten material is relatively insufficient. Decreasing LT weakens the staircase effect, promotes full spreading of the melt tracks and filling of inter-filament gaps, reduces surface undulations, and thus decreases Ra. A thin LT tends to cause edge squeezing and overflow of melt tracks, forming irregular protrusions and local material accumulation that increases microscopic surface unevenness and Ra.

In Figure 6b, the BC (ER-ID) interaction has a significant effect on Ra (*p* = 0.0425). As ER increased from 0.9 to 1.0, the variation in Ra with ID changed from gentle to significantly increasing: at low ER, the effect of ID on Ra was weak, and Ra remained at a relatively low level overall; at high ER, Ra significantly increased with decreasing ID, reaching a peak at ID = 20%. This observation is similar to the results recorded in Ref. [49]. At low ER, material supply is relatively constrained, and the surface profile is mainly governed by microscopic depressions from insufficient material deposition (rather than incomplete melting), while ID weakly modulates surface undulations. At high ER, the material fill is abundant; high ID suppresses melt track shrinkage and collapse through internal support, forming regular filament ridges and a gentle profile on the surface; at low ID, insufficient support makes the surface prone to defects such as collapse, warpage and disorder, significantly increasing Ra.

### 3.3. Analysis of the Relationship Between Ra and COF from BBD Experimental Results

**Figure 7:**
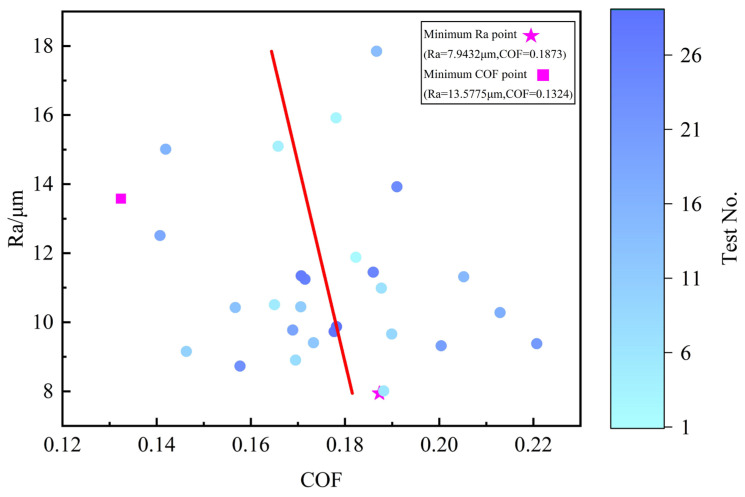
Correspondence and linear fitting between Ra and COF of specimens.

To quantitatively reveal the coupling relationship between surface roughness Ra and friction coefficient COF of FDM-printed ABS samples and quantify their mutual performance trade-off under single-objective optimisation, this section analyses all 29 groups of BBD experimental data via correlation statistics and performance comparison. The scatter distribution of measured Ra and COF values for each test group is displayed in Figure 7, where each data point corresponds to one experimental run numbered 1–29. Test No. 1 obtained the minimum surface roughness with Ra = 7.9432 μm and COF = 0.1873, while Test No. 22 achieved the lowest friction coefficient with COF = 0.1324 and Ra = 13.5775 μm. The linear fitting relationship between the two indicators (the red line in Figure 7) is expressed as Equation (5):

(5) $$Ra=\frac{1}{0.0017}\left(0.1953-COF\right)$$

Pearson linear correlation [50] and Spearman rank correlation [51] analysis are adopted to evaluate the correlation between Ra and COF from linear and rank perspectives, respectively. The rank transformation calculation for Spearman correlation follows the standard nonparametric statistical procedure described in reference. Statistical results showed that the Pearson correlation coefficient between Ra and COF was −0.2038 (*p* = 0.2889 > 0.05), and the Spearman rank correlation coefficient was −0.1754 (*p* = 0.3629 > 0.05). Both *p*-values exceeded 0.05, indicating no statistically significant linear correlation existed between Ra and COF within the selected process parameter range. There was neither an obvious linear trend nor fixed monotonic change rule between the two indicators. This result indicates that the linear correspondence—that improving specimen surface quality necessarily leads to simultaneous improvement in friction and frictional performance—does not hold for FDM-printed ABS components.

Comparative analysis of single-objective optimisation results revealed obvious mutual restriction between Ra and COF. In Figure 7, when minimising Ra was set as the sole optimisation target, the optimal Ra reached 7.9432 μm, but the corresponding COF rose to 0.1873, which was 41.47% higher than the global minimum COF value and seriously deteriorated the wear resistance. Conversely, if only COF was optimised to the minimum value of 0.1324, the matched Ra increased sharply to 13.5775 μm, representing a 70.93% degradation in surface smoothness. These quantified data directly prove that optimising only one performance indicator will inevitably cause severe attenuation of the other. Traditional single-objective optimisation strategies fail to meet the comprehensive service demands of FDM functional parts because they cannot balance low surface roughness and low friction simultaneously. The weak correlation and non-linear variation characteristics of Ra and COF further demonstrate that linear fitting alone cannot predict performance evolution accurately. Hence, multi-objective intelligent optimisation combined with response surface quadratic models is required to break the inherent performance trade-off limitation and provide parametric matching guidance for fabricating FDM ABS components with both excellent surface quality and friction performance.

## 4. Multi-Objective Optimisation and Discussion

**Figure 8:**
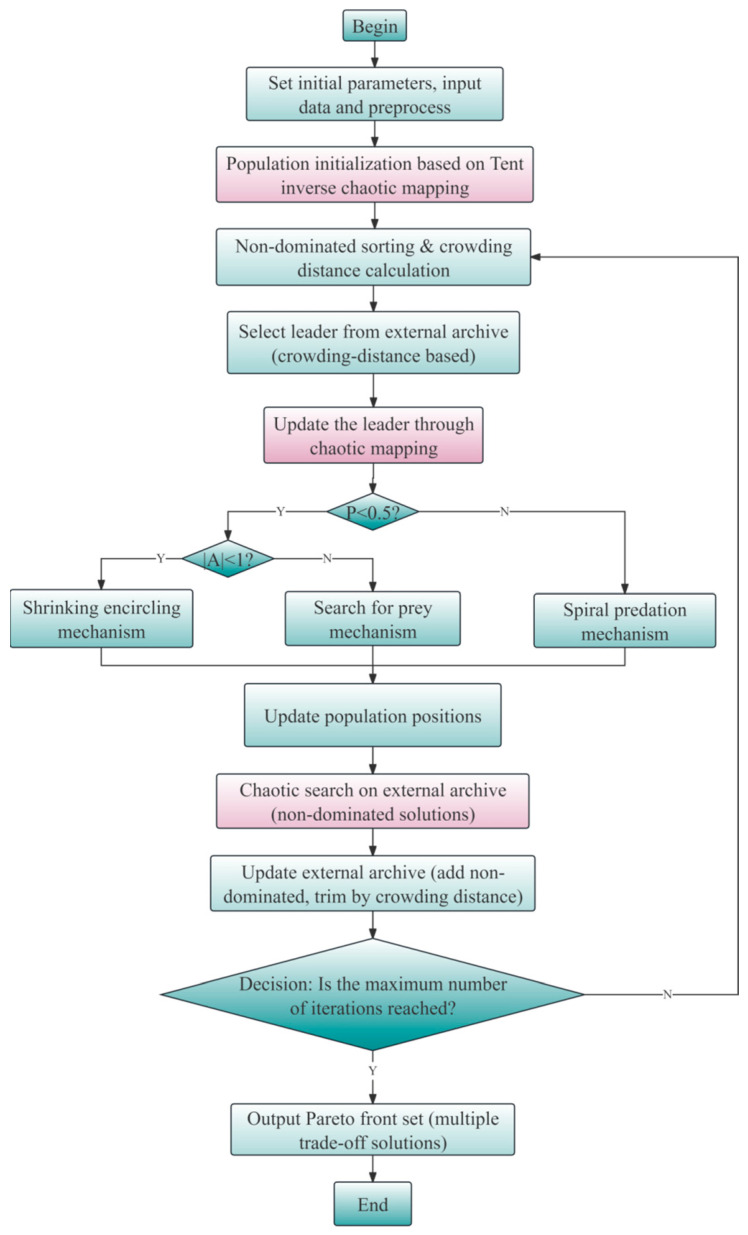
Flowchart of the CWOA model.

The chaotic whale optimisation algorithm (CWOA), based on the standard whale optimisation algorithm (WOA), incorporates chaotic opposition-based learning, a co-updating strategy of convergence factor and inertia weight with non-linear chaotic perturbation, and chaotic search for the optimal individual to reduce the probability of becoming trapped in local optima [52,53]. The flowchart of the CWOA is shown in Figure 8, and its distinctive features are indicated by filled red boxes in Figure 8.

The population based on Tent inverse mapping, *OX* = {*OX_id_*, *d* = *1*, *2*, *…*, *D*; *i* = *1*,*2*, *…*, *N*}, where each individual OXid is expressed as:

*OX_id_* = *X_mind_* + *X_maxd_* − *X_id_* (6)

where X_id_ is the d-dimensional code value of the i-th population. X_mind_ and X_maxd_ are the lower and upper search bounds of X_id_.

In multi-objective optimisation, the Pareto dominance relation is adopted: for any two solutions *u* and *v*, if *f_m_*(*u*) ≤ *f_m_*(*v*) for all objectives m and there exists at least one objective for which the inequality holds strictly, then *u* is said to dominate *v*. To maintain uniformity of the Pareto front, the crowding distance *CD*_i_ of each non-dominated solution is calculated using Equation (7):

(7) $$CD_{i}=\sum_{m=1}^{M}\frac{f_{m}\left(i+1\right)-f_{m}\left(i-1\right)}{f_{m}^{\max}-f_{m}^{\min}}$$

where *M* is the number of objectives.

During the initialisation, the initial population is subjected to non-dominated sorting, and an external archive (storing all non-dominated solutions) is initialised. In the main loop, at each iteration, the solution with the largest crowding distance is first selected from the external archive as the leader *X*_leader_. The leader is then updated using the logistic chaotic map: z_new_ = 4*z*(1 − *z*) to generate chaotic variables, which are then mapped back to the original search space. If the new solution dominates the original solution, it replaces it.

The predation mechanism mainly includes shrinking encircling, random search and spiral updating. The execution of predation behaviour depends on the probability *p* and the coefficient vector *A*, calculated as follows:

(8) $$X^{t+1}=\left\{\begin{array}{c} \omega \cdot X_{leader}^{t}-A\cdot \left|C\cdot X_{leader}^{t}-\left.X^{t}\right|\begin{array}{cc} & p<0.5,\left|A\right|<1 \end{array}\right.\\ X_{rand}^{t}-A\cdot \left|C\cdot X_{rand}^{t}-\left.X^{t}\right|\begin{array}{cc} & p<0.5,\left|A\right|\geq 1 \end{array}\right.\\ \omega \cdot X_{leader}^{t}+D\cdot e^{bl}\cdot cos\left(2\pi l\right)\begin{array}{cc} & p\geq 0.5 \end{array} \end{array}\right.$$

where $X^{t}$ is the position vector of the current whale individual; $X_{leader}^{t}$ is the leader selected from the external archive; D is the distance between the whale and the leader during spiral updating; $X_{rand}^{t}$ is the position vector of a randomly selected whale individual; *A* and *C* are coefficients, with *A* = 2*a*·*r*_1_ − *a*, C = 2*r*_2_; the convergence factor *a* linearly decreases from 2 to 0 over iterations; *r*_1_ and *r*_2_ are random numbers in [0, 1]; *p* is a random number in [0, 1] representing the probability of executing predation behaviour; *w* is the inertia weight (typically following a non-linear decreasing strategy); *b* is a constant defining the shape of the logarithmic spiral (typically taken as 1); and *l* is a random number in [−1, 1].

After updating the positions of all individuals, a chaotic local search is performed for each non-dominated solution in the external archive to further approximate the true Pareto front. For each non-dominated solution in the archive (denoting the d-th dimension of the k-th non-dominated solution in the archive as *X_kd_*), it is mapped to the interval −1, 1 using Equation (9). A chaotic sequence $y_{kd}^{t_{c}}$ is then generated iteratively using Equation (10). Finally, a new position vector $X_{kd}^{t_{c}}$ is generated by carrier wave back to the neighbourhood of the original search space using Equation (11). If the new solution dominates the original solution, it replaces it, and the external archive is re-maintained.

(9) $$y_{kd}=\frac{2\left(X_{kd}-X_{mind}\right)}{X_{maxd}-X_{mind}}-1$$

(10) $$y_{kd}^{tc}=1-2{\left(y_{kd}^{t_{c}-1}\right)}^{2}$$

(11) $$X_{kd}^{t_{c}}=X_{mind}+\frac{X_{maxd}-X_{mind}}{2}\left(y_{kd}^{t_{c}}+1\right)$$

where $y_{kd}^{t_{c}}\in \left(-1,1\right),t_{c}=1,2,\ldots ,t_{cmax}$, *t_cmax_* is the maximum number of iterations for chaotic search.

Finally, the current population and the external archive are merged, non-dominated sorting and crowding distance calculation are performed again, and the external archive is updated (all non-dominated solutions are added; if the archive exceeds capacity, it is trimmed in descending order of the crowding distance). If the maximum number of iterations is not reached, the process returns to reselect the leader from the external archive and continues the loop; otherwise, the Pareto front solution set from the external archive is output.

With the aim of simultaneously reducing the surface COF and Ra of specimens, a set of optimal process parameters was sought. The boundary conditions for each experimental factor and the objectives are as follows:

(12) $$\left\{\begin{array}{l} \begin{array}{c} Z_{\mathrm{COF}}=\left(A_{i},B_{i},C_{i},D_{i}\right)\to \min\\ Z_{\mathrm{Ra}}=\left(A_{i},B_{i},C_{i},D_{i}\right)\to \min \end{array}\\ s.t.\left\{\begin{array}{l} 0.16\;\mathrm{mm}\leq A_{i}\leq 0.24\;\mathrm{mm}\\ 0.9\leq B_{i}\leq 1\\ 20\%\leq C_{i}\leq 100\%\\ 230\;{}^{\circ}C\leq D_{i}\leq 270\;{}^{\circ}C \end{array}\right. \end{array}\right.$$

**Figure 9:**
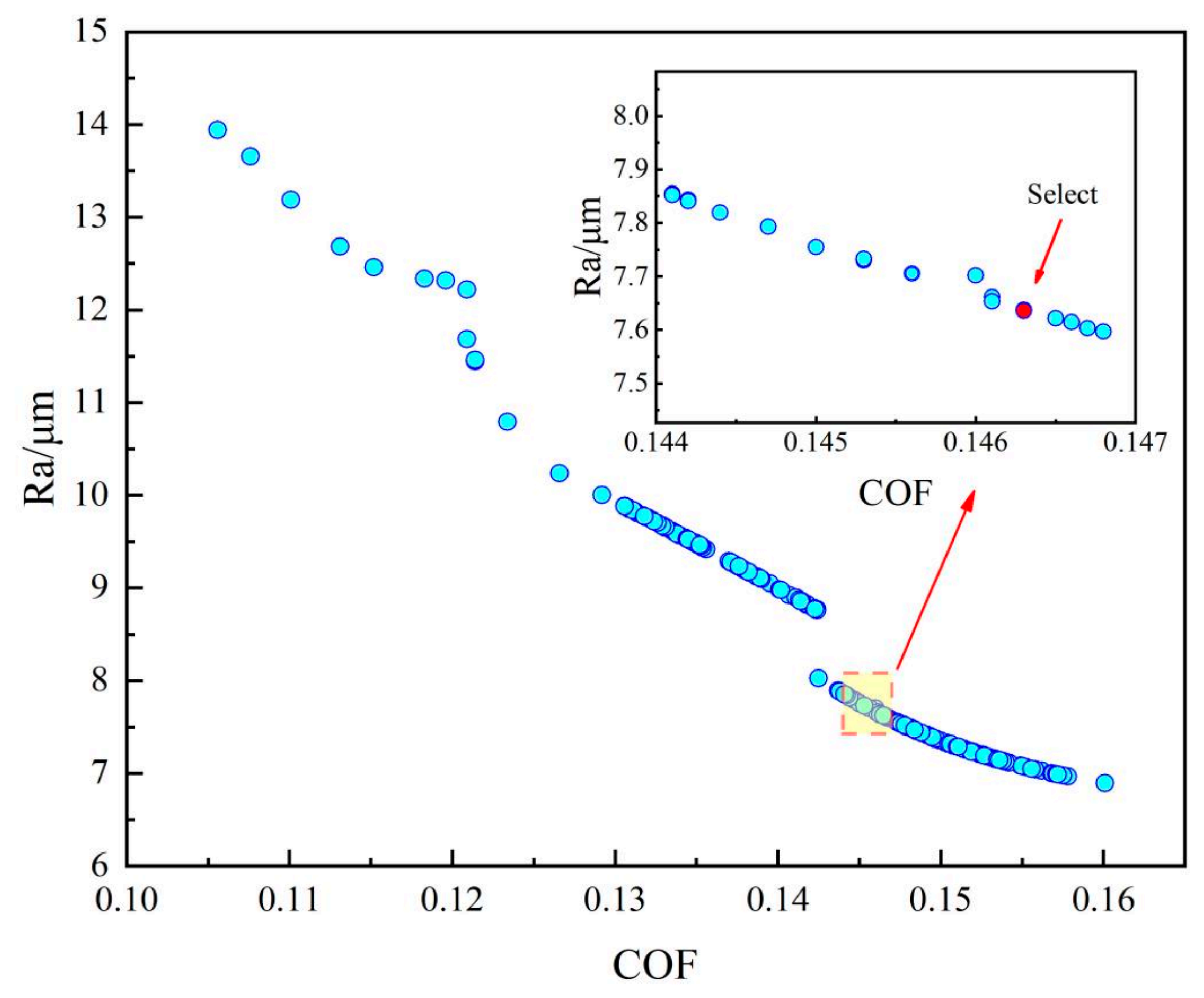
Pareto front set of optimisation results.

**Figure 10:**
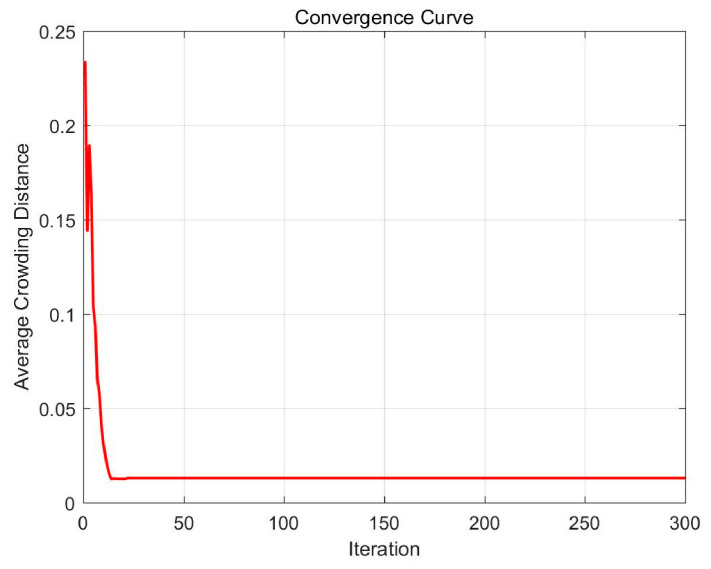
Convergence analysis.

Referring to the recommended population range of 200–500 for the standard CWOA in Ref. [32] and considering the convergence characteristics of the present non-dominated solution problem, a preliminary experiment was conducted with an initial population size of 300 and a maximum iteration number of 300. Figure 9 exhibits the Pareto front set obtained from the optimisation runs. Figure 10 presents the convergence curve of the optimisation algorithm. The average crowding distance declined sharply at the early iterations, and the algorithm achieved rapid convergence at approximately the 30th generation. The crowding distance remained stable without obvious fluctuations from the 30th to the 300th generation, which demonstrates favourable convergence stability of the algorithm. The TOPSIS method was used to comprehensively evaluate the multi-objective optimisation results. Vector normalisation was performed on COF and Ra to eliminate dimensional effects. Both objectives were assumed equally important, each assigned a weight of 0.5. A positive ideal solution was constructed with minimum COF and minimum Ra, and a negative ideal solution was constructed in the opposite direction. The comprehensive closeness coefficient for each solution was calculated; the closer the closeness coefficient to 1, the better the comprehensive performance of the solution.

**Figure 11:**
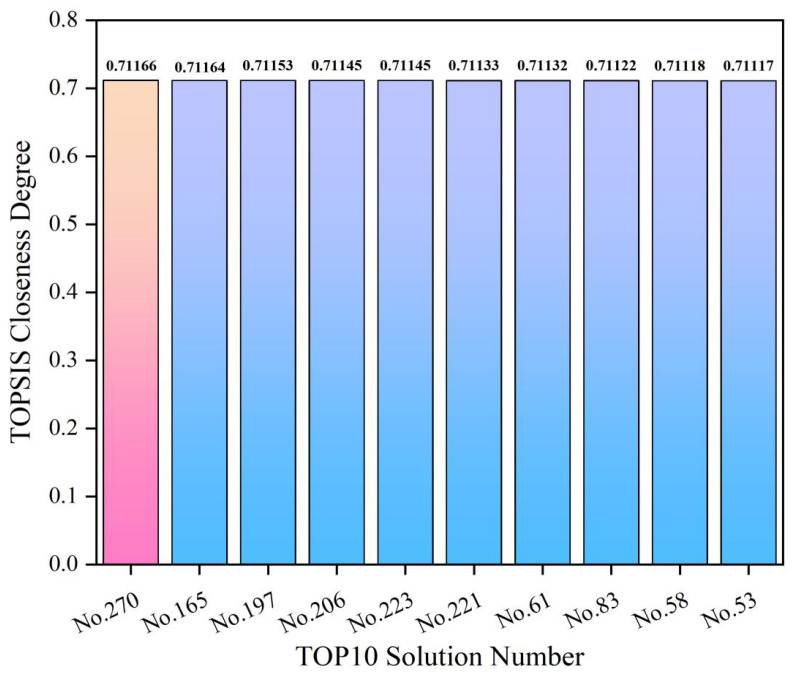
Closeness coefficient comparison of top 10 solutions.

**Figure 12:**
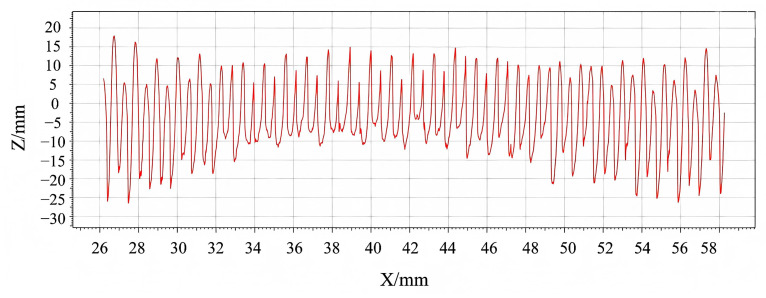
Roughness curve of the optimal specimen.

**Figure 13:**
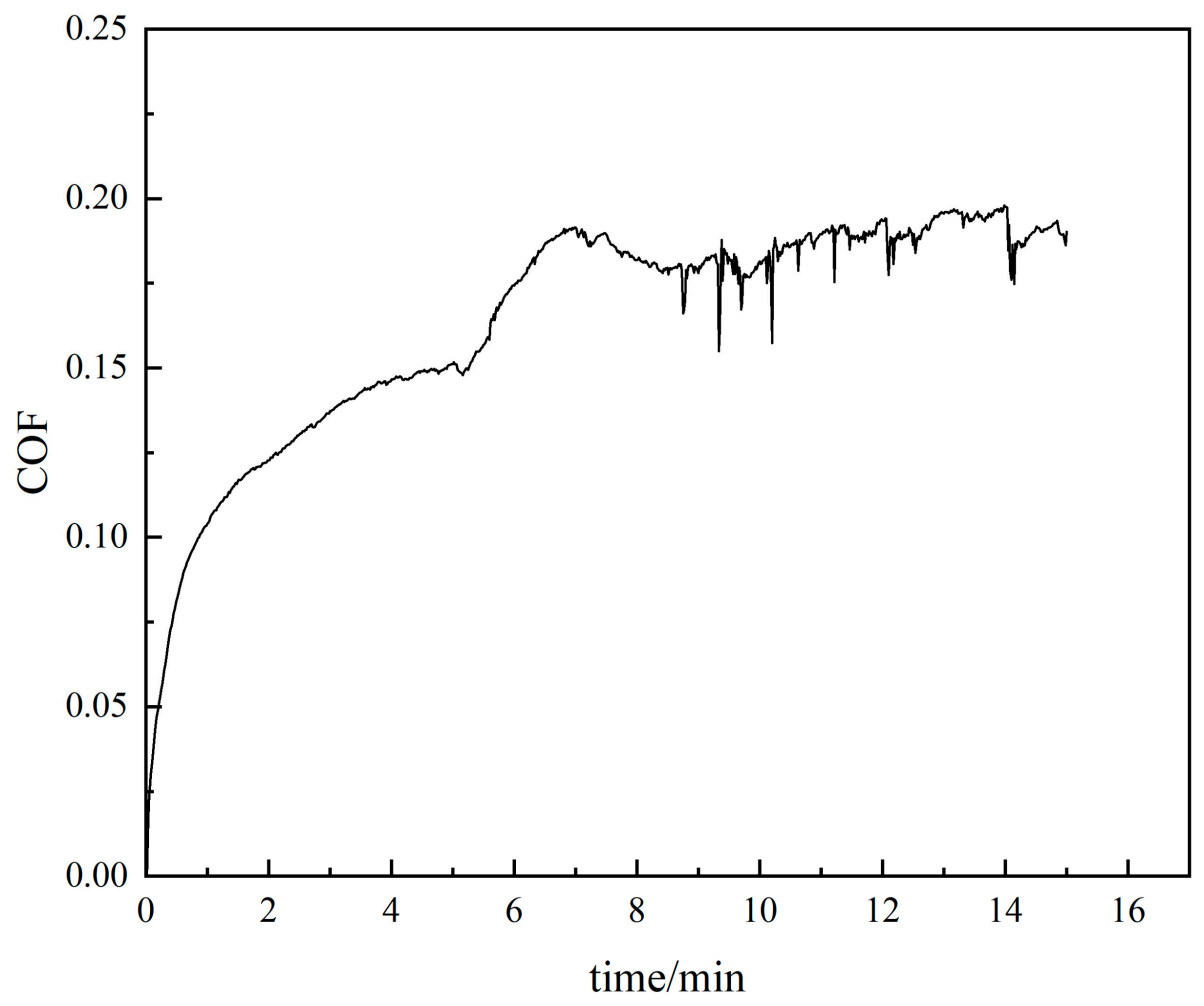
COF test curve of the optimal specimen.

Separate specimens were fabricated under the optimal process parameters determined via multi-objective CWOA optimisation for experimental verification. Table 4 lists the specific process parameters and predicted response values of the top 10 Pareto non-dominated solutions ranked by the TOPSIS method. Figure 11 compares the closeness coefficients of these top 10 Pareto non-dominated solutions evaluated by TOPSIS. Among all candidates, Solution 270 exhibited the maximum closeness coefficient of 0.71166 and was thus selected as the optimal parameter combination. Figure 12 presents the surface roughness profile curve of the optimal specimen, which exhibited uniform and mild surface undulations without prominent sharp peaks or deep valleys. Figure 13 illustrates the time-varying COF curve recorded during the friction test of the optimal specimen. Following the initial running-in stage, the friction coefficient rapidly stabilised within a narrow range, demonstrating steady frictional performance.

Table 5 quantitatively compares the predicted performance values and experimental measurements of the optimal specimen. Each specimen was subjected to three replicate tests. The average measured Ra reached 7.7921 μm, with a relative error of only 2.04% compared to the predicted value of 7.6360 μm; the average measured COF was 0.1504, corresponding to an error of 2.80% relative to the predicted value of 0.1463. All prediction errors are less than 5%, which verifies the high prediction accuracy of the established quadratic regression models and the reliability of the CWOA multi-objective optimisation strategy.

To quantitatively evaluate the optimisation effect and effectively validate the synergistic optimisation capability for surface quality and friction performance, the average of five repeated central-point trials (Runs 25–29) with medium process parameters was selected as the benchmark reference, and the comparative results are presented in Table 6. The baseline values of Ra and COF were 10.7293 μm and 0.1768, respectively, while the measured average values of Ra and COF for the optimal specimen were 7.7921 μm and 0.1504. Compared with the baseline, the optimal specimen achieved synchronous reductions in both Ra and COF, with Ra decreased by 27.4% and COF reduced by 14.9%. It should be clarified that the Pareto optimal compromise solution cannot independently attain the absolute global minimum of either individual objective. Even so, the synchronous reduction in both response indicators relative to the baseline fully confirms the synergistic optimisation capability of the proposed framework.

Combining the BBD response surface experimental results, typical ABS 3D-printed specimens and the multi-objective optimised optimal specimen were selected for surface metallographic morphology observation. The results are shown in Figure 13. The bright yellow regions in the figures were extruded filament ridges, and the dark bands were inter-filament valleys between adjacent filaments. Due to the light-trapping effect of surface depressions, the inter-filament valley regions appear black.

**Figure 14:**
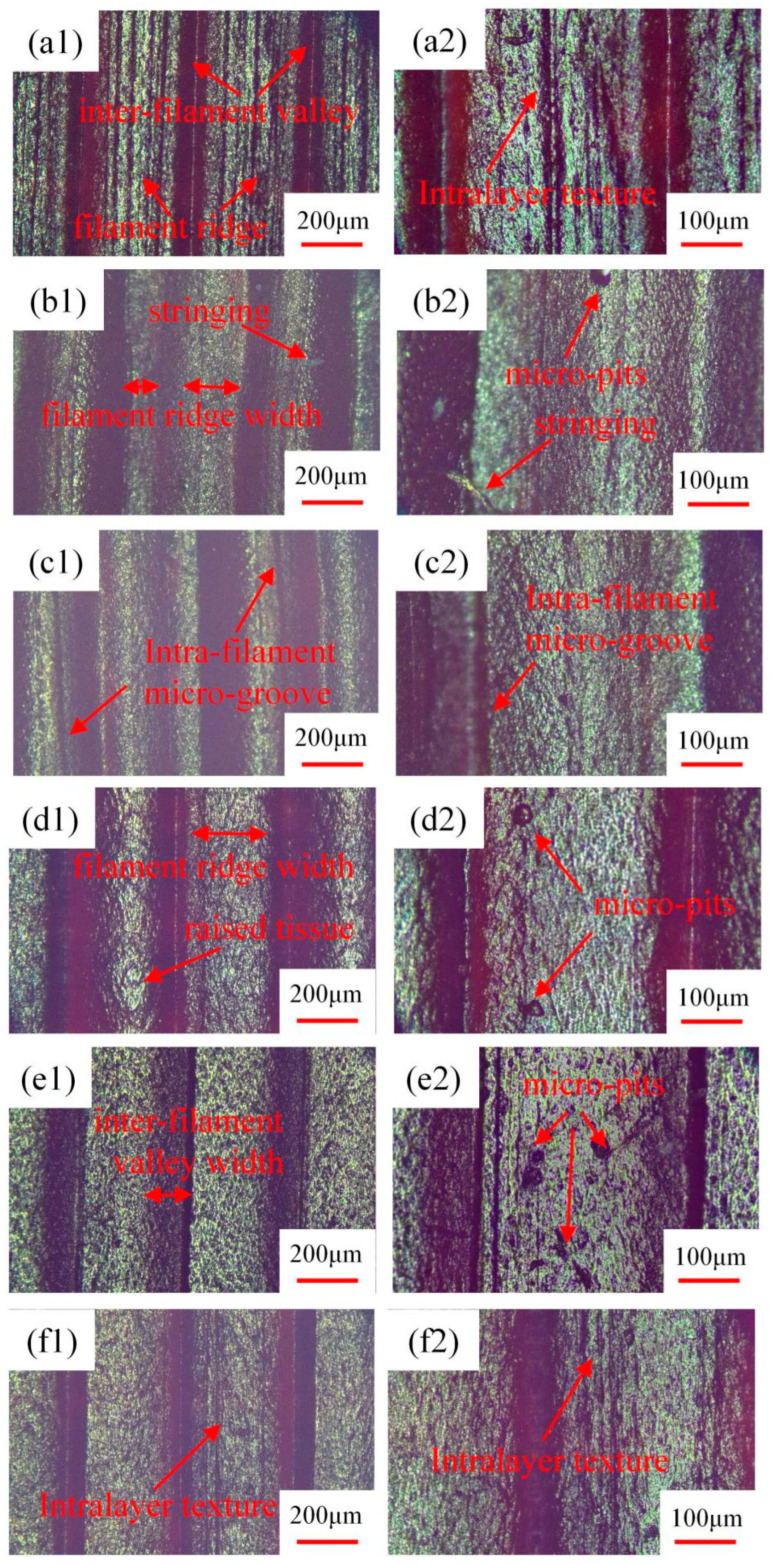
Surface morphology of specimens. (**a1**,**a2**) Test No. 1; (**b1**,**b2**) Test No. 14; (**c1**,**c2**) Test No. 22; (**d1**,**d2**) Test No. 21; (**e1**,**e2**) Test No. 27; (**f1**,**f2**) optimal specimen.

Figure 14(a1,a2) shows the metallographic morphology of specimen No. 1 (Ra = 7.9432 μm, COF = 0.1873). The specimen exhibited a periodic printing texture, with relatively large filament ridge spacing and regular, narrow inter-filament valleys. This regular peak-and-valley structure gave it the lowest Ra among the BBD experimental groups. The clear contrast between light and dark regions indicates a large surface height difference. The hard, protruding filament ridges may tend to cause ploughing of the counterpart surface, and the fine intralayer textures further intensify cutting wear, ultimately resulting in a high COF.

Figure 14(b1,b2) shows the metallographic morphology of specimen No. 14 (Ra = 17.8511 μm, COF = 0.1867). This specimen had densely distributed and skewed inter-filament valleys, with frequent surface profile undulations, accompanied by forming defects such as stringing and micro-pits, which gave it the largest Ra among the BBD experimental groups. Compared with specimen No. 1, the contrast between light and dark regions was lower, indicating a smaller surface height difference. However, the disordered surface defects exacerbate friction interface wear, also leading to a high COF.

Figure 14(c1,c2) shows the metallographic morphology of specimen No. 22 (Ra = 13.5775 μm, COF = 0.1324). The dense arrangement of filament ridges was the main reason for the relatively high Ra. The surface grooves were regular in morphology, the overall profile undulations are gentle, the colour difference between light and dark patterns is weak, and there are no obvious forming defects. The blunted intra-filament texture may weaken the ploughing effect, while intra-filament micro-grooves can accommodate wear debris and reduce the actual contact area of the interface, significantly lowering frictional resistance. Consequently, this specimen had a relatively high Ra but the lowest COF among the BBD experimental groups.

Figure 14(d1,d2) shows the metallographic morphology of specimen No. 21 (Ra = 9.3793 μm, COF = 0.2207). This specimen had wide filament ridges and large inter-filament valley spacing, with gentle overall profile fluctuations, resulting in moderate roughness. However, the wide ridges increase the frictional contact area and intensify interfacial adhesion. Combined with surface raised tissues and micro-pits defects, this tends to cause interfacial bonding and material transfer during friction, may lead to severe adhesive wear. This feature gave this specimen the highest COF among the BBD experimental groups.

Figure 14(e1,e2) shows the metallographic morphology of BBD centre point specimen No. 27 (Ra = 11.3451 μm, COF = 0.1707). The filament ridge width, profile undulations, and spacing were moderate; surface defects such as micro-pits and intralayer textures were relatively balanced. Consequently, both Ra and COF were at moderate levels among the BBD experimental groups.

Figure 14(f1,f2) shows the metallographic morphology of the optimal specimen (Ra = 7.7921 μm, COF = 0.1463) obtained from multi-objective optimisation. The surface microstructure was uniform and dense, profile undulations were significantly improved, filament ridges were wide, inter-filament valleys were narrow, and Ra was lower than those of all BBD experimental specimens. Moreover, the excellent melt forming quality substantially reduces various surface defects, leaving only a small amount of intralayer texture, effectively reducing frictional resistance and achieving synergistic optimisation of surface roughness and frictional performance.

In summary, Ra and COF of FDM-printed ABS specimens are jointly influenced by filament ridge arrangement, inter-filament valley morphology and forming defects. Surface defects such as micro-pits, stringing, and disordered textures simultaneously exacerbate surface roughness and frictional loss. However, the responses of Ra and COF to micro-morphology differ significantly: Ra is primarily dominated by the macroscopic peak–valley structure and the density of filament ridges, while COF may be controlled by the ploughing effect, actual contact area, and groove debris storage capacity. Therefore, simply reducing roughness cannot achieve a simultaneous decrease in COF; it is necessary to consider filament ridge size, groove structure and forming defect control to achieve multi-objective synergistic optimisation of Ra and COF.

## 5. Conclusions

Quadratic regression models for surface roughness (Ra) and coefficient of friction (COF) of FDM-printed ABS parts were established using a Box–Behnken design. The models were highly significant (*p* < 0.0001), with R^2^ values of 0.9421 and 0.9474, respectively. ANOVA showed that extrusion ratio (ER) had a highly significant effect on Ra (F = 120.08, *p* < 0.0001), while LT and ER had significant effects on COF (*p* < 0.001). The highly significant interaction terms (*p* < 0.01) for COF were AC (LT-ID), AD (LT-ET), BC (ER-ID), BD (ER-ET) and CD (ID-ET), and the significant interaction effects (*p* < 0.05) for Ra were AB (LT-ER) and BC (ER-ID).

The Pearson correlation coefficient between Ra and COF was −0.2038 (*p* = 0.2889 > 0.05), and the Spearman rank correlation coefficient was −0.1754 (*p* = 0.3629 > 0.05), indicating no significant linear correlation between the two within the experimental parameter range. Single-objective optimisation results showed that when Ra minimisation was the sole objective, the optimal Ra = 7.9432 μm corresponded to a COF of 0.1873, which was 41.47% higher than the global minimum COF. When COF minimisation was the sole objective, the optimal COF = 0.1324 corresponded to a Ra of 13.5775 μm, which was 70.93% higher than the global minimum Ra. These results confirm a clear trade-off constraint between surface quality and frictional performance, necessitating multi-objective synergistic optimisation.

Based on the Pareto-dominated chaotic whale optimisation algorithm (CWOA) combined with TOPSIS comprehensive evaluation, the optimal process parameter combination was obtained: LT 0.16 mm, ER 0.9, ID 66%, ET 270 °C. Under these conditions, the predicted Ra was 7.6360 μm and the measured Ra was 7.7921 μm (relative error 2.04%); the predicted COF value was 0.1463, while the measured value was 0.1504 (relative error 2.80%). In addition, the average values of the five repeated central-point trials from the BBD were selected as the benchmark reference. Compared with the baseline, the optimised samples achieved synchronous reduction in Ra and COF (Ra decreased by 27.4%, COF decreased by 14.9%). These results validate the accuracy of the prediction model and the effectiveness of the collaborative optimisation strategy implemented by the CWOA.

Metallographic morphology analysis revealed that according to the pre-wear morphology, Ra of FDM-printed ABS parts was primarily controlled by the macroscopic peak–valley structure and filament ridge density, whereas COF may be mainly affected by the ploughing effect, actual contact area, and intra-filament groove debris storage capacity. Surface defects such as micro-pits, stringing, and disordered textures simultaneously exacerbate surface roughness and frictional loss. The optimal specimen surface exhibited wide filament ridges, regular and narrow inter-filament valleys, and an absence of micro-pits and stringing defects, which was the micro-mechanism enabling synergistic improvement of Ra and COF.

The RSM-CWOA synergistic optimisation framework proposed in this paper provides a generalisable approach for multi-objective process parameter optimisation in FDM additive manufacturing, with prediction errors below 5%. Future work can extend this method to other polymer material systems (e.g., PLA, PETG), incorporate additional process parameters (e.g., printing speed, build orientation), and validate the service life of optimised parts through long-term friction and wear testing.

## Figures and Tables

**Table 1:** Experimental factors and levels.

Run	Factors			
	A (LT)/mm	B (ER)	C (ID)/%	D (ET)/°C
−1	0.16	0.9	20	240
0	0.2	0.95	60	255
1	0.24	1	100	270

**Table 2:** Design parameters and results.

STD	A (LT)/mm	B (ER)	C (ID)/%	D (ET)/)/°C	COF	Ra/μm
1	0.16	0.90	60	255	0.1873	7.9432
2	0.24	0.90	60	255	0.1823	11.8797
3	0.16	1.00	60	255	0.1781	15.9189
4	0.24	1.00	60	255	0.1658	15.0974
5	0.20	0.95	20	240	0.1650	10.5084
6	0.20	0.95	100	240	0.1882	8.0112
7	0.20	0.95	20	270	0.1877	10.9873
8	0.20	0.95	100	270	0.1695	8.9052
9	0.16	0.95	60	240	0.1899	9.6585
10	0.24	0.95	60	240	0.1463	9.1572
11	0.16	0.95	60	270	0.1706	10.4458
12	0.24	0.95	60	270	0.1733	9.4078
13	0.20	0.90	20	255	0.1567	10.4279
14	0.20	1.00	20	255	0.1867	17.8511
15	0.20	0.90	100	255	0.2052	11.3164
16	0.20	1.00	100	255	0.1419	15.0125
17	0.16	0.95	20	255	0.2129	10.2795
18	0.24	0.95	20	255	0.1407	12.5109
19	0.16	0.95	100	255	0.1689	9.7755
20	0.24	0.95	100	255	0.2004	9.3182
21	0.20	0.90	60	240	0.2207	9.3793
22	0.20	1.00	60	240	0.1324	13.5775
23	0.20	0.90	60	270	0.1577	8.7327
24	0.20	1.00	60	270	0.1910	13.9256
25	0.20	0.95	60	255	0.1860	11.4510
26	0.20	0.95	60	255	0.1715	11.2458
27	0.20	0.95	60	255	0.1707	11.3451
28	0.20	0.95	60	255	0.1777	9.7319
29	0.20	0.95	60	255	0.1782	9.8729

**Table 3:** Analysis of variance (ANOVA).

Source	COF			Ra		
	F-Value	*p*-Value	Significant	F-Value	*p*-Value	Significant
Model	18.03	<0.0001	Yes	16.28	<0.0001	Yes
A	17.79	0.0009	Yes	1.34	0.2663	
B	23.63	0.0003	Yes	120.08	<0.0001	Yes
C	1.08	0.3158		12.49	0.0033	Yes
D	0.0969	0.7602		0.5330	0.4774	
AB	0.2907	0.5982		8.11	0.0129	Yes
AC	58.66	<0.0001	Yes	2.59	0.1298	
AD	11.69	0.0041	Yes	0.1032	0.7527	
BC	47.49	<0.0001	Yes	4.98	0.0425	Yes
BD	80.66	<0.0001	Yes	0.3546	0.5610	
CD	9.35	0.0085	Yes	0.0618	0.8073	
A^2^	0.0176	0.8964		0.3419	0.5680	
B^2^	0.1373	0.7165		47.79	<0.0001	Yes
C^2^	0.2265	0.6415		0.5682	0.4635	
D^2^	1.01	0.3317		15.21	0.0016	
Lack of fit	1.28	0.4374	Not	0.9493	0.5721	Not
R^2^		0.9474			0.9421	
Adjusted R^2^		0.8949			0.8843	
Predicted R^2^		0.7498			0.7387	
Adeq precision		16.3903			15.1560	
C.V.%		3.85			7.48	

**Table 4:** Top 10 process parameters and response prediction values.

Solution Ranking	No.	A	B	C	D	COF	Ra
1	270	0.16	0.9	65.95	270	0.1463	7.6360
2	165	0.16	0.9	66.73	270	0.1461	7.6541
3	197	0.16	0.9	66.05	270	0.1463	7.6383
4	206	0.16	0.9	64.54	270	0.1467	7.6037
5	223	0.16	0.9	65.34	270	0.1465	7.6219
6	221	0.16	0.9	65.04	270	0.1466	7.6151
7	61	0.16	0.9	64.25	270	0.1468	7.5971
8	83	0.16	0.9	67.04	270	0.1461	7.6615
9	58	0.16	0.9	70.94	270	0.1450	7.7554
10	53	0.16	0.9	68.89	270	0.1456	7.7055

**Table 5:** Comparison of predicted and measured results after optimisation.

Response	Predicted Value	Repeated Measured Values	Mean Measured Value	Standard Deviation	Error/(%)
Z_Ra_/μm	7.6360	7.7321, 7.8168, 7.8274	7.7921	0.0522	2.04
Z_COF_	0.1463	0.1533, 0.1504, 0.1475	0.1504	0.0029	2.80

**Table 6:** Performance comparison between the multi-objective optimal specimen and baseline central-point specimen.

Response	Runs 25–29	Baseline (Average of Runs 25–29)	Optimal Solution (*n* = 3 Mean)	Reduction Rate/(%)
Z_Ra_/μm	11.4510, 11.2458, 11.3451, 9.7319, 9.8729	10.7293	7.7921	27.4
Z_COF_	0.1860, 0.1715, 0.1707, 0.1777, 0.1782	0.1768	0.1504	14.9

## Data Availability

The data presented in this study are openly available in [Testing and optimisation of 3D-printed ABS parts] [10.5281/zenodo.20763493], accessed on 19 June 2026.
